# Elemental sulfur concentration can be used as a rapid, reliable, and cost-effective predictor of sulfur amino acid content of soybean seeds

**DOI:** 10.1038/s41598-024-53590-3

**Published:** 2024-02-07

**Authors:** Wonseok Kim, Sunhyung Kim, Thomas P. Mawhinney, Hari B. Krishnan

**Affiliations:** 1 Division of Plant Science and Technology, University of Missouri, Columbia, MO 65211 USA; 2 Department of Biochemistry, University of Missouri, Columbia, MO 65211 USA; 3 Plant Genetics Research Unit, USDA, Agricultural Research Service, University of Missouri, 108 Curtis Hall, Columbia, MO 65211 USA

**Keywords:** Proteins, Plant physiology

## Abstract

In this study, we have examined the feasibility of using elemental sulfur content of soybean seeds as a proxy for the overall sulfur amino acid content of soybean seeds. Earlier, we have identified by high throughput ionomic phenotyping several high and low sulfur containing soybean lines from the USDA Soybean Germplasm Collection. Here, we measured the cysteine and methionine content of select soybean lines by high-performance liquid chromatography. Our results demonstrate that those soybean lines which had high elemental sulfur content also had a higher cysteine and methionine content when compared to soybean lines with low elemental sulfur. SDS-PAGE and immunoblot analysis revealed that the accumulation of Bowman Birk protease inhibitor and lunasin in soybean seeds may only be marginally correlated with the elemental sulfur levels. However, we found a positive correlation between the levels of trypsin and chymotrypsin inhibitor activities and elemental sulfur and sulfur amino acid content of the seeds. Thus, elemental sulfur content and/or protease inhibitor activity measurement can be utilized as a rapid and cost-effective method to predict the overall sulfur amino acid content of soybean seeds. Our findings will benefit breeders in their endeavors to develop soybean cultivars with enhanced sulfur amino acid content.

## Introduction

Soybeans, which are processed to yield both meal and oil (http://ncsoy.org/media-resources/uses-of-soybeans/), accumulate about 36–40% protein and 18–20% oil in their seeds^1^. Importantly, soybean meal serves as the world's largest source of animal protein feed and is looked upon as “gold standard” to which other protein sources are compared^2^. However, the nutritive quality of soybeans could be further improved by enhancing the concentrations of sulfur-containing amino acids (methionine and cysteine). Methionine is an essential amino acid, while cysteine is considered as “conditionally” essential since animals can synthesize this amino acid from methionine^3^. Methionine plays a key role in numerous cellular functions while cysteine serves as an important structural and functional component of proteins and enzymes^3^. Due to their crucial functions both methionine and cysteine are indispensable for human and animal nutrition^4^. Consequently, feed lacking adequate quantities of these amino acids will negatively impact the livestock growth and development.

Methionine and cysteine content of soybean seeds is about 1.4%, which is not adequate to meet the monogastric animal nutritional needs^5^. To overcome this deficiency, animal feeds are supplemented with synthetic amino acids which adds cost to the animal feed. Hence, the United Soybean Board’s Better Bean Initiative (BBI) and poultry industry has emphasized the importance of increasing the sulfur containing amino acid content of soybean from the current level of 1.4–2.1% (http://soybeaninnovationlab.illinois.edu/files/PoultrySoybeanUse.pdf). Efforts have been made in recent decades to increase the levels of cysteine and methionine in soybean seeds^6–10^. Both classical breeding and genetic engineering approaches have been used to increase sulfur-containing amino acid levels with varying degrees of success^5,11^. Soybean breeders have identified quantitative trait loci (QTL) and candidate alleles associated with methionine and cysteine concentrations^12–14^. Despite these advances, challenges remain in developing elite soybean varieties with improved sulfur-containing amino acid content. One of the challenges faced by soybean breeders is the lack of a rapid, inexpensive, and reliable method for the measurement of methionine and cysteine content in large numbers of soybean seed samples.

Currently, methods used to quantify these amino acids in soybeans are time-consuming and cost prohibitive. High-performance liquid chromatography (HPLC) and more recently hydrophilic interaction chromatography coupled tandem mass spectrometry have been used for precise amino acid quantification^15,16^. However, these methods are time-consuming and expensive and limit their use in plant-breeding efforts that require rapid screening large numbers of samples. Near-infrared (NIR) spectroscopy and Handheld Near-Infrared Sensor have also been employed for rapid measurement of amino acids in soybean seeds^17–21^. A recent study evaluated the use of energy-dispersive X-ray fluorescence (XRF) sensor to classify soybeans based on their protein content. The authors demonstrated that sulfur signals and other XRF spectral features can be utilized as proxies to infer soybean protein content^22^. Though NIR spectroscopy is amenable for high-throughput screening, this procedure has limitations in precisely quantifying levels of sulfur amino acids.

Ionomics involves the quantitative and simultaneous measurement of the mineral nutrient and trace element composition of living organisms^23^. High throughput elemental profiling has been employed to identify soybean lines that accumulate high and low elemental sulfur^24,25^. Inductively Coupled Plasma Mass Spectroscopy (ICP-MS) analysis of the elemental profile of seeds from 1653 lines in the USDA Soybean Germplasm Collection led to the identification of several soybean lines accumulating high elemental sulfur^25^. However, this study did not examine if these high elemental sulfur containing soybean lines also reveal high sulfur amino acid content. Since the ICP-MS is well suited for high-throughput screening and provides robust elemental profile, its utility in predicting the sulfur amino acid of soybean seeds will be a valuable tool for soybean breeders in their endeavors to develop soybean cultivars with enhanced sulfur amino acid content. In this study, we have selected several high sulfur accumulators identified from the USDA Soybean Germplasm Collection to elucidate their amino acid composition by HPLC. Our results demonstrate that those soybean lines which had high sulfur content also had a higher cysteine and methionine content when compared to soybean lines with low elemental sulfur. Based on our results we propose that elemental sulfur determination can be used as a reliable predictor of sulfur amino acid content of soybean seeds. We also examined if any correlation exists between elemental sulfur content and the accumulation of sulfur-rich proteins (leginsulin, Bowman-Birk protease inhibitor, and 2S albumin) in soybean seeds. In general, we found seeds with higher sulfur content accumulated relatively higher levels of Bowman-Birk protease inhibitor and higher protease inhibitor activity when compared to soybean lines with lower sulfur content.

## Results and discussion

Earlier, we analyzed the elemental profile of seeds from 1653 PI lines in the USDA Soybean Germplasm Collection^25^. Based on this analysis, several PI lines were grouped into either as low (less than 3000 ppm) or high (greater than 3000 ppm) sulfur accumulating lines. Residual seeds from our earlier study^25^ was also used for the current investigation. For this study, we selected eight high sulfur accumulating lines and four low sulfur accumulating lines. The PI number, maturity group, origin, percent protein and oil and sulfur content of these PI lines are shown in Table 1. All the PI lines used in study had their origin from East Asia except PI437377 which originated from Primorye, a Far East region of Russia. The protein content ranged from 40 to 51%. Due to the known negative correlation between protein and oil content, PI lines with greater than 40% protein content revealed much lower oil content (Table 1). The sulfur content in high sulfur accumulating lines ranged from 3886 to 5043 ppm, while in the low sulfur accumulating lines it ranged between 2780 and 2842 ppm (Table 1). The elemental profile of these soybean PI lines remained almost similar even when these soybean lines were grown for several years in various environments, whether in the field or the greenhouse. Soybean PI lines that were initially identified as high and low sulfur accumulating lines consistently retained this trait, indicating sulfur content is intrinsic to the genetics of the PI lines^25^.

**Table 1:** Relevant characteristics of soybean lines used in this study.

PI Number	Maturity group^a^	Origin^a^	Seed color^a^	% Protein^a^	% Oil^a^	Elemental sulfur (ppm)^b^
PI603162	IV	North Korea	Black	50.2	14.5	3901.58
PI603910B	IV	North Korea	Black	51.1	14.0	3987.43
PI549045A	IV	China	Black	50.6	8.2	5042.88
PI437377	III	USSR	Brown	49.6	15.3	3885.86
PI468919	III	China	Black	45.3	12.3	4530.75
PI082278	III	South Korea	Black	46.2	13.2	4070.29
PI339734	IV	South Korea	Black	50.4	10.9	3999.77
PI424078	III	South Korea	Black	47.4	12.2	4402.74
PI096322	III	North Korea	Black	40.8	21.9	2783.31
PI229327	IV	Japan	Yellow	40.4	18.7	2780.23
PI507411	IV	Japan	Yellow	40.6	19.8	2833.45
PI603599A	IV	China	Yellow	44.5	15.8	2842.30

^a^Data compiled from USDA-ARS Germplasm Resources Information Network (USDA-ARS, 2010).

^b^Data compiled from Ziegler et al.^25^.

### Is there a correlation between elemental sulfur and sulfur amino acid content?

**Figure 1:**
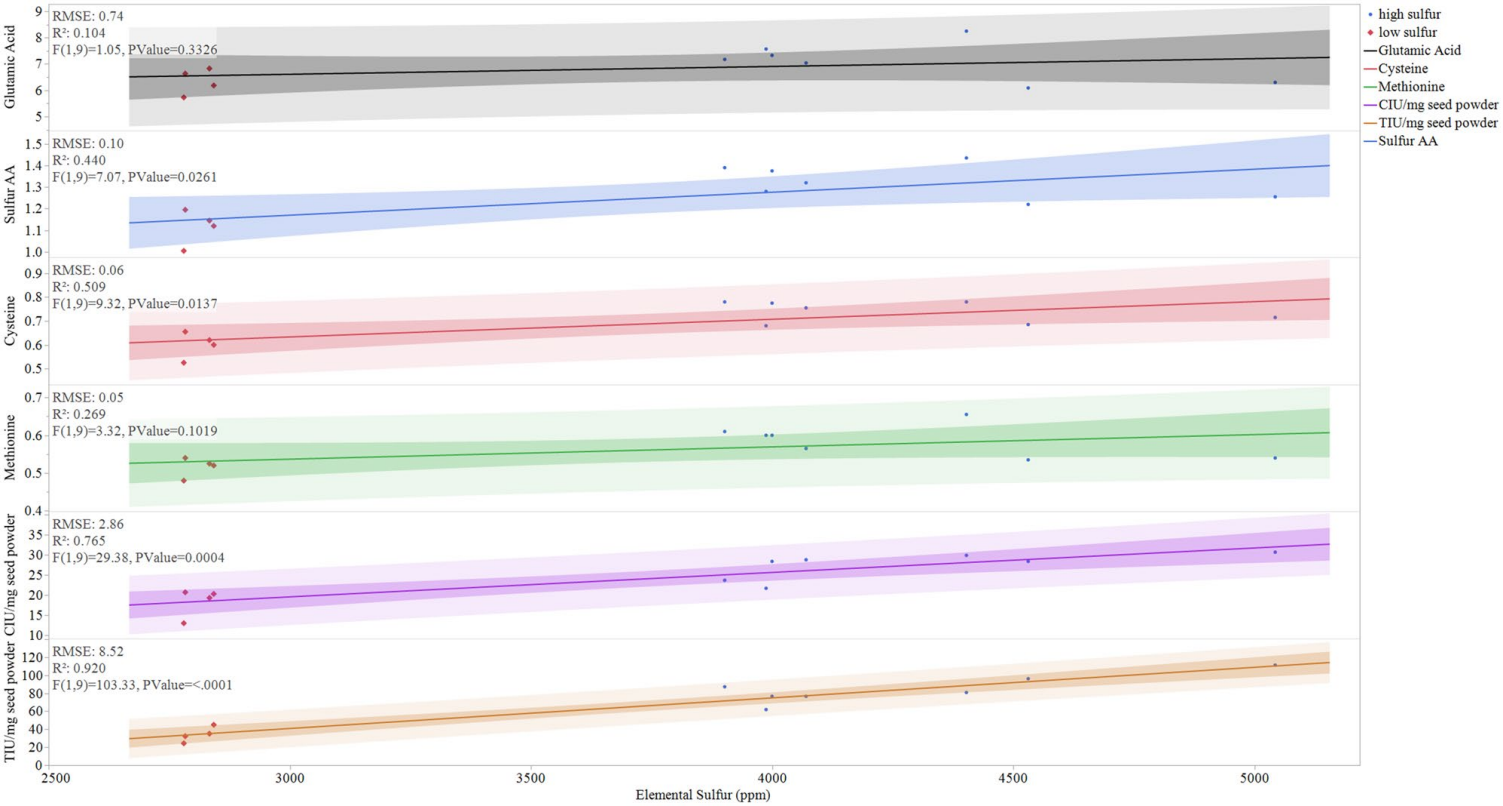
Relationships between total seed elemental sulfur content and other seed components. RMSE indicates root mean square deviation, R2 indicates the coefficient of determination, F indicates the F-value and *P*-value indicates the output of the statistical test for correlation. The protease inhibitor activities are expressed as TIU (trypsin units inhibited) and CIU (chymotrypsin units inhibited) per milligram seed powder.

To examine if there is a correlation between elemental sulfur and sulfur amino acid content of seeds, we employed HPLC to measure the amino acid content of the high and low sulfur accumulating lines (Table 2). The total amino acid content (Methionine + Cysteine) of soybean lines with high elemental sulfur was higher when compared with soybean lines with low elemental sulfur content (Table 2). Among the high sulfur lines, PI424078 and PI437377 exhibited the highest sulfur amino acid content with 1.4%. The total amino acid content of low-sulfur lines ranged from 1.0 to 1.2%. The average total sulfur content of high sulfur soybean lines was 1.3% while the average total sulfur amino acid content of low sulfur lines was 1.1%. We examined if there was any correlation exists between elemental sulfur content and the individual amino acids namely aspartic acid, threonine, serine, glutamic acid, proline, glycine, alanine, cysteine, valine, methionine, isoleucine, leucine, tyrosine, phenylalanine, lysine, histidine, and arginine. We applied the FitYbyX function of JMP16 to look for correlations between sulfur content with amino acid to graph linear trends between these data (Fig. 1). We observed a significant relationship between seed elemental sulfur content and cysteine content (r2 = 0.509, *P*-value = 0.0173). A weak correlation was observed between methionine content and elemental sulfur content (r2 = 0.269, *P*-value = 0.1019). However, a significant correlation was found between the sum of the Cys and Met amino acids (Sulfur AA) and elemental sulfur (r2 = 0.440, *P*-value = 0.0261). No significant correlations were identified between sulfur content and any other amino acids (e.g., Glutamic acid, r2 = 0.104, *P*-value = 0.326).

**Table 2:** High Performance Liquid Chromatography measurement of amino acid content of soybean Plant Introduction lines (% per 100 g protein).

Amino acid^a^	PI603162 (Mean ± SD)	PI603910B (Mean ± SD)	PI549045A (Mean ± SD)	PI437377 (Mean ± SD)	PI468919 (Mean ± SD)	PI082278 (Mean ± SD)	PI339734 (Mean ± SD)	PI424078 (Mean ± SD)	PI096322 (Mean ± SD)	PI229327 (Mean ± SD)	PI507411 (Mean ± SD)	PI603599A (Mean ± SD)
Aspartic Acid	4.58 ± 0.25	4.83 ± 0.06	4.04 ± 0.23	5.08 ± 0.08	3.95 ± 0.40	4.58 ± 0.06	4.68 ± 0.17	5.26 ± 0.11	4.23 ± 0.13	3.63 ± 0.02	4.31 ± 0.21	4.02 ± 0.26
Threonine	1.47 ± 0.07	1.59 ± 0.04	1.30 ± 0.08	1.62 ± 0.01	1.35 ± 0.14	1.43 ± 0.04	1.55 ± 0.06	1.67 ± 0.04	1.46 ± 0.06	1.24 ± 0.01	1.46 ± 0.08	1.38 ± 0.08
Serine	1.67 ± 0.09	1.73 ± 0.05	1.47 ± 0.10	1.83 ± 0.03	1.48 ± 0.16	1.64 ± 0.02	1.70 ± 0.08	1.84 ± 0.08	1.52 ± 0.06	1.31 ± 0.01	1.56 ± 0.10	1.44 ± 0.09
Glutamic Acid	7.18 ± 0.42	7.57 ± 0.04	6.30 ± 0.34	8.11 ± 0.16	6.09 ± 0.64	7.04 ± 0.09	7.33 ± 0.33	8.25 ± 0.20	6.64 ± 0.25	5.73 ± 0.03	6.83 ± 0.33	6.19 ± 0.40
Proline	2.00 ± 0.15	2.14 ± 0.03	1.81 ± 0.04	2.26 ± 0.09	1.75 ± 0.16	2.04 ± 0.01	2.13 ± 0.16	2.29 ± 0.15	1.93 ± 0.12	1.63 ± 0.01	1.92 ± 0.09	1.70 ± 0.10
Glycine	1.67 ± 0.10	1.74 ± 0.01	1.49 ± 0.08	1.83 ± 0.03	1.51 ± 0.16	1.64 ± 0.03	1.73 ± 0.08	1.88 ± 0.05	1.57 ± 0.02	1.37 ± 0.01	1.61 ± 0.05	1.50 ± 0.06
Alanine	1.63 ± 0.09	1.72 ± 0.01	1.43 ± 0.08	1.77 ± 0.02	1.46 ± 0.16	1.62 ± 0.05	1.69 ± 0.07	1.82 ± 0.04	1.59 ± 0.05	1.39 ± 0.01	1.61 ± 0.08	1.51 ± 0.09
Cysteine	0.78 ± 0.03	0.68 ± 0.01	0.72 ± 0.05	0.80 ± 0.00	0.69 ± 0.06	0.76 ± 0.01	0.78 ± 0.04	0.78 ± 0.00	0.66 ± 0.04	0.53 ± 0.01	0.62 ± 0.03	0.60 ± 0.03
Valine	1.97 ± 0.10	2.17 ± 0.01	1.75 ± 0.09	2.23 ± 0.04	1.77 ± 0.20	2.00 ± 0.03	2.10 ± 0.09	2.28 ± 0.05	1.97 ± 0.08	1.69 ± 0.01	2.00 ± 0.09	1.84 ± 0.11
Methionine	0.61 ± 0.03	0.60 ± 0.00	0.54 ± 0.03	0.64 ± 0.01	0.54 ± 0.05	0.57 ± 0.01	0.60 ± 0.03	0.66 ± 0.01	0.54 ± 0.03	0.48 ± 0.00	0.53 ± 0.04	0.52 ± 0.03
Isoleucine	1.92 ± 0.10	2.04 ± 0.02	1.66 ± 0.09	2.15 ± 0.04	1.67 ± 0.18	1.89 ± 0.04	1.96 ± 0.09	2.14 ± 0.05	1.86 ± 0.07	1.63 ± 0.01	1.95 ± 0.09	1.76 ± 0.11
Leucine	2.87 ± 0.15	3.13 ± 0.04	2.53 ± 0.13	3.25 ± 0.04	2.55 ± 0.29	2.86 ± 0.06	2.98 ± 0.12	3.26 ± 0.09	2.81 ± 0.11	2.43 ± 0.00	2.91 ± 0.14	2.64 ± 0.17
Tyrosine	1.32 ± 0.06	1.43 ± 0.02	1.16 ± 0.07	1.47 ± 0.01	1.20 ± 0.13	1.32 ± 0.08	1.38 ± 0.06	1.50 ± 0.01	1.30 ± 0.04	1.11 ± 0.00	1.32 ± 0.06	1.23 ± 0.08
Phenylalanine	1.94 ± 0.10	2.10 ± 0.03	1.72 ± 0.09	2.22 ± 0.03	1.73 ± 0.19	1.98 ± 0.02	2.05 ± 0.09	2.24 ± 0.06	1.93 ± 0.08	1.72 ± 0.00	2.05 ± 0.10	1.82 ± 0.11
Lysine	2.58 ± 0.16	2.79 ± 0.01	2.35 ± 0.12	2.86 ± 0.04	2.38 ± 0.25	2.62 ± 0.01	2.70 ± 0.11	2.94 ± 0.06	2.49 ± 0.07	2.19 ± 0.01	2.54 ± 0.11	2.39 ± 0.13
Histidine	1.03 ± 0.06	1.13 ± 0.01	0.99 ± 0.05	1.13 ± 0.01	0.96 ± 0.10	1.07 ± 0.01	1.14 ± 0.05	1.28 ± 0.03	1.02 ± 0.03	0.87 ± 0.01	1.02 ± 0.05	0.94 ± 0.06
Arginine	2.91 ± 0.16	3.64 ± 0.06	2.94 ± 0.19	3.26 ± 0.03	2.54 ± 0.28	3.36 ± 0.04	3.31 ± 0.13	4.18 ± 0.10	2.71 ± 0.10	2.48 ± 0.02	2.75 ± 0.13	2.69 ± 0.21

^a^Data are the average of two independent measurements and error for each value was calculated as the standard deviation of the mean.

### Do high sulfur containing soybean lines accumulate higher amounts of sulfur-rich proteins?

**Figure 2:**
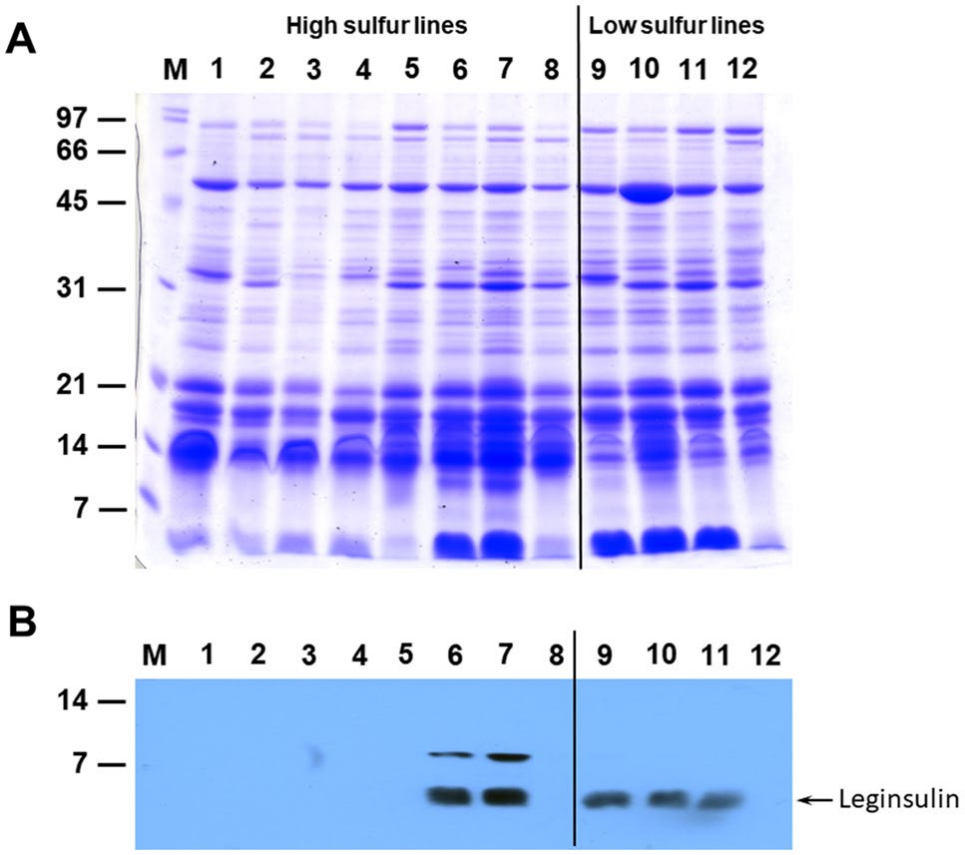
Panel A: SDS-PAGE analysis of 50% isopropanol-extracted proteins from mature soybean seed. Equal amounts of dry soybean seed powder were extracted with 50% isopropanol and the recovered proteins were resolved on 15% SDS-PAGE gels. Proteins were visualized by staining with Coomassie Brilliant Blue. Panel B: Immunological detection of leginsulin in soybean seeds. Isopropanol-extracted proteins were first separated on a 15% SDS-PAGE and were electrophoretically transferred to nitrocellulose membranes and probed with soybean leginsulin antibodies. Immunoreactive proteins were detected using anti rabbit immunoglobulin G (IgG)–horseradish peroxidase conjugate followed by chemiluminescent detection. The exposure time was 30 s. An empty lane between high sulfur and low sulfur lines was deleted and merged (indicated with a black dividing line). Complete uncropped digital images used for creating (**A**) and (**B**) are included in the supplemental file. Lane 1, PI603162; lane 2, PI603910B; lane 3, PI549045A; lane 4, PI437377; lane 5, PI468919; lane 6, PI082278; lane 7, PI339734; lane 8, PI424078; lane 9, PI096322; lane 10, PI229327; lane 11, PI507411; lane 12, PI603599A. The position and sizes of protein markers in kilodaltons are shown at the left of the figure.

In addition to the abundant 11S and 7S globulins, soybeans also accumulate a limited number of proteins that are enriched in sulfur amino acids^26,27^. Leginsulin is a sulfur-rich protein that accumulates in soybean seeds. Soybean leginsulin is synthesized as a precursor polypeptide that is subjected to posttranslational processing to give rise to 6 kDa and 4 kDa subunits^28,29^. The 4 kDa subunit is named leginsulin and is enriched in cysteine residues^28^. We have previously reported that leginsulin predominantly accumulates in Asian soybean cultivars and were mostly absent in North American cultivars^30^. We examined the accumulation of this cysteine-rich protein among soybean lines differing in sulfur content. For this purpose, we extracted 50% isopropanol soluble proteins which has been shown to preferentially extract leginsulin^30^. Figure 2 shows the Coomassie Blue stained protein profile of high and low sulfur accumulating soybean PI lines extracted with 50% isopropanol. A 4 kDa protein, which has been previously identified as leginsulin^30^, was seen to be abundantly present in only a few soybean lines. Western blot analysis using purified soybean leginsulin antibodies revealed the accumulation of a 4 kDa protein only in PI082278, PI339734, PI096322, PI229327, and PI507411. Leginsulin accumulation was not detected in other soybean lines (Fig. 2). PI082278 and PI339734 belong to the high sulfur content group, while PI096322, PI229327, and PI507411 were grouped under low sulfur content group. Based on this observation, there is no stringent correlation between the elemental sulfur content and the accumulation of leginsulin.

**Figure 3:**
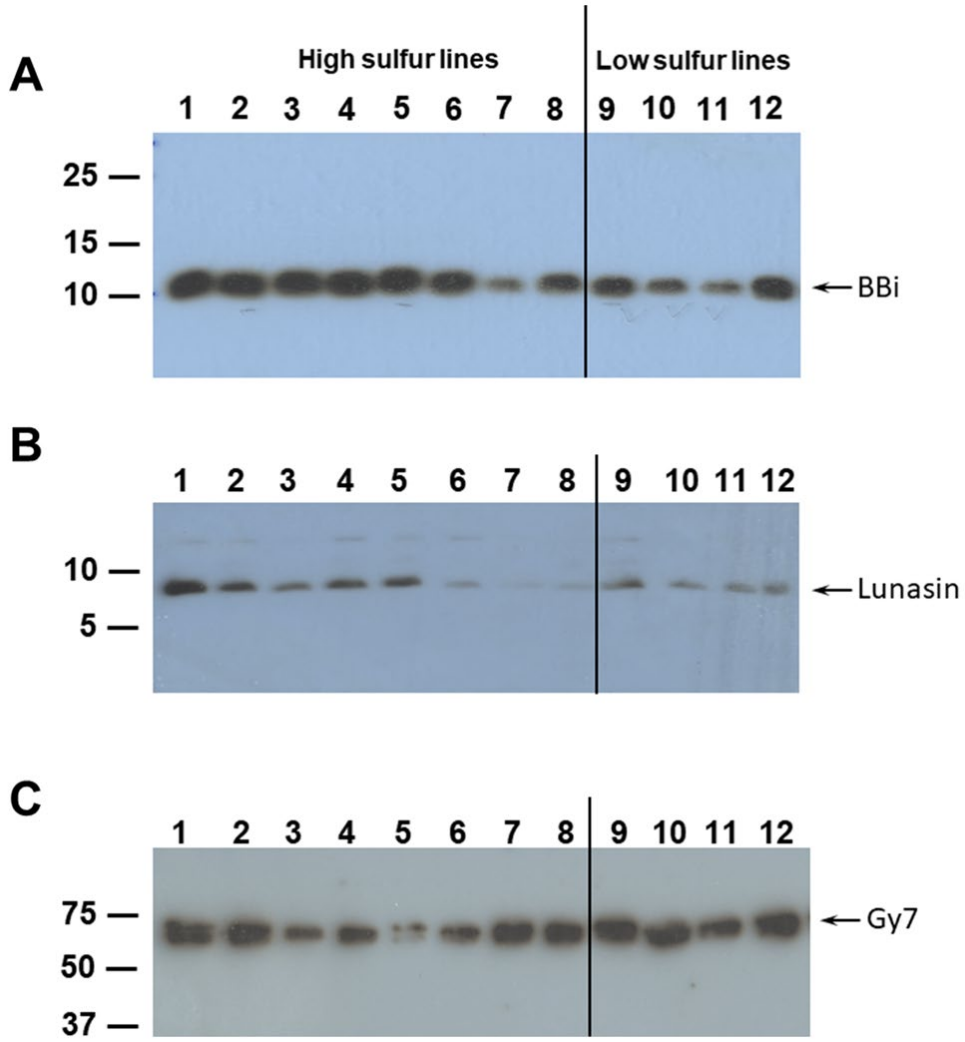
Immunological detection of Bowman-Birk protease inhibitor, lunasin and glycinin in soybean seeds. Total seed proteins (50 µg) resolved by 15% SDS-PAGE were transferred to nitrocellulose membrane and incubated with affinity purified soybean Bowman-Birk protease inhibitor antibody (Panel A) or lunasin (Panel B) or glycinin Gy7 (Panel C). Immunoreactive proteins were visualized by using antirabbit IgG horseradish peroxidase conjugate antibody followed by chemiluminescent detection. The exposure time was 30, 120, and 20 s for Bowman-Birk protease inhibitor, lunasin, and glycinin Gy7 detection, respectively. Complete uncropped digital images used for creating (**A**), (**B**), and (**C**) are included in the supplemental file. Lane 1, PI603162; lane 2, PI603910B; lane 3, PI549045A; lane 4, PI437377; lane 5, PI468919; lane 6, PI082278; lane 7, PI339734; lane 8, PI424078; lane 9, PI096322; lane 10, PI229327; lane 11, PI507411; lane 12, PI603599A. The position and sizes of protein markers in kilodaltons are shown at the left of the figure.

Another prominent sulfur-rich protein in soybean is the Bowman-Birk protease inhibitor. This protein has been suggested to be a major contributor of the total sulfur content in soybean seeds^31–33^. They contain a high proportion of cysteine residues which results in the formation of seven disulfide bridges^32,33^. We wanted to examine if the soybean lines with high elemental sulfur content accumulate higher levels of the Bowman-Birk protease inhibitors. For this purpose, we performed western blot analysis using Bowman-Birk protease inhibitor specific peptide antibodies raised in rabbits^34^. The peptide antibodies specifically reacted against an 11–12 kDa polypeptide. In general, a stronger reaction with BBi-specific antibodies was detected in six out eight soybean extracts that had higher elemental sulfur content (Fig. 3A). This protein also accumulated in soybean lines with low sulfur content, though the intensity of the immunoreactive bands was slightly lower than the lines with higher sulfur content (Fig. 3A). The accumulation of Bowman-Birk protease inhibitor in PI339734 and PI424078 was marginally lower than other soybean lines with high sulfur content (Fig. 3A). However, we cannot exclude the possibility that these soybean lines may accumulate other isoforms of BBI which may not be recognized by the antibodies used in our study. In this regard it should be pointed out that at least 10 BBI isoforms exist in soybean seed, which have been shown to have different specific inhibitory activities on trypsin, chymotrypsin, and/or elastase^35,36^. We also examined differences in the accumulation of 2S albumin, a methionine-rich protein. The 2S albumin is synthesized as a precursor protein and contains a signal peptide, a methionine-rich 8 kDa larger subunit, an aspartic acid-rich 6 kDa small subunit corresponding to lunasin^37,38^. Lunasin, a 43-amino acid peptide, has been shown to have anti-cancer and anti-inflammatory properties^39,40^. Immunoblot analysis using peptide antibodies raised against lunasin^41^ reacted against a low molecular weight protein whose accumulation was marginally higher in soybean PI lines with higher sulfur amino acid content (Fig. 3B). As an internal loading control, we utilized antibodies raised against a 11S glycinin (Gy7). This antibody detected a 62 kDa polypeptide in all soybean lines both with high and low sulfur content (Fig. 3C). We also noticed that the accumulation of this protein also showed slight variation among the different soybean PI lines. Our findings show that the accumulation of BBi and lunasin in soybean seeds may only be slightly correlated with the elemental sulfur level.

### Soybean lines with high sulfur content reveal higher levels of protease inhibitor activity

Since our study showed relatively higher amount of BBI accumulation in soybean lines with high sulfur content, we wanted to examine if there is any correlation between BBI activity and sulfur content. Soybeans contain two major protease inhibitors: the Kunitz trypsin inhibitor and the Bowman-Birk protease inhibitor^26,27,31^. Soybean BBI are capable of inhibiting both trypsin and chymotrypsin and hence we measured both trypsin and chymotrypsin inhibitor activities of seed extract from the high and low sulfur containing soybean lines. Table 3 shows the trypsin and chymotrypsin inhibitor activity of the soybean seed samples. Interestingly, soybean lines with high elemental sulfur had significantly higher levels of chymotrypsin inhibitor activity when compared to lines with lower sulfur content. Their activity ranged between 35.15 and 21.75 CIU/mg seed powder (Table 3). Among high sulfur soybean lines, PI437377 had the highest activity while soybean line PI603910B had the lowest level (21.75 CIU/mg seed powder). In contrast, low sulfur-containing soybean lines had much lower chymotrypsin inhibitor activity. Their activity ranged between 12.98 and 20.65 CIU/mg seed powder (Table 3).

**Table 3:** Trypsin and chymotrypsin inhibitor activity of soybean Plant Introduction lines used in this study^a^

PI number	Trypsin inhibitor activity TIU/mg seed powder	Chymotrypsin inhibitor Activity CIU/mg seed powder
Williams 82	65.60 ± 1.94	22.55 ± 0.54
PI603162	87.33 ± 4.54	23.68 ± 0.76
PI603910B	61.89 ± 6.98	21.75 ± 0.65
PI549045A	111.28 ± 2.41	30.73 ± 0.23
PI437377	110.7 ± 0.93	35.15 ± 1.43
PI468919	96.22 ± 1.34	28.43 ± 0.63
PI082278	76.72 ± 1.90	28.75 ± 0.61
PI339734	76.72 ± 1.42	28.37 ± 0.59
PI424078	80.89 ± 1.39	29.92 ± 1.65
PI096322	32.28 ± 1.78	20.65 ± 0.71
PI229327	24.39 ± 1.11	12.98 ± 0.34
PI507411	35.06 ± 0.25	19.34 ± 0.08
PI603599A	45.00 ± 2.17	20.31 ± 0.75

^a^Trypsin and chymotrypsin inhibitor activity were measured under standard conditions with *N*-benzoyl-l-arginine ethyl ester and *N*-benzoyl-l-tyrosine ester as substrates, respectively. *TIU* trypsin units inhibited, *CIU* chymotrypsin units inhibited.

Since Bowman-Birk protease inhibitors not only inhibit chymotrypsin but also trypsin, we measured the trypsin inhibitor activity from the high and low sulfur containing soybean lines (Table 3). Trypsin inhibitor activity in high-sulfur soybean lines was markedly higher than in lower sulfur soybean lines. Trypsin inhibitor activity in high sulfur lines ranged from 62 to 111 TIU/mg seed powder. PI549045A and PI437377 had significantly higher trypsin inhibitor activity with 111 and 110 TIU/mg seed powder (Table 3). Low sulfur soybean lines showed drastically lower trypsin inhibitor activity when compared with the high sulfur containing lines. The TI activity in low sulfur lines ranged between 24 and 45 TIU/mg seed powder. We observed a significant relationship (Fig. 1) between seed elemental sulfur content and chymotrypsin inhibitor activity (r2 = 0.765, *P*-value = 0.0004) and between sulfur content and trypsin inhibitor activity (r2 = 0.920, *P*-value < 0.0001). We also noted strong positive relationships between cysteine and chymotrypsin inhibitor activity (r2 = 0.72; *P*-value 0.0119) and trypsin inhibitor activity (r2 = 0.71; *P*-value 0.0150).

In this study, we have revealed that elemental sulfur content of soybean seed can be used as a proxy to rapidly identify soybean lines with relatively higher sulfur amino acid content. This trait is important for improving the nutritional quality and health benefits of soybean products. Ionomics is amenable to high throughput screening and is cost-effective, so breeders could widely exploit this method as a method of choice to rapidly identify soybean lines with high sulfur amino acid content. However, breeders previously used near-infrared reflectance spectroscopy to evaluate this trait in segregating soybean populations. The accuracy of this procedure is questionable, and calibration flaws in the instruments could affect the outcomes. Therefore, we propose high throughput ionomic phenotyping as a rapid and reliable method to predict the sulfur amino acid content of soybean seed. We also demonstrated that soybean lines with high elemental sulfur content not only had higher sulfur amino acid content but also expressed higher levels of trypsin and chymotrypsin inhibitor activities**.** Our observation suggests that soybean PI lines with higher elemental sulfur content may accumulate higher amounts of protease inhibitors in their seeds. However, additional experiments are required to establish a strong correlation between the availability of increased sulfur amino acids and the accumulation of sulfur-rich proteins.

## Materials and methods

### Seed material and plant growth

Residual soybean seeds from our previous study^25^ was utilized for our current study. Soybean plant introduction (PI) lines examined in this study and their cultivation in the field has also been described earlier^25^. Briefly, soybean PI lines were grown in small plots at Bradford Research and Extension Center, Columbia, Missouri. High and low sulfur accumulating soybean PI lines were grown in small plots at Bradford Research and Extension Center, Columbia, Missouri. Cultural practices were typical of those utilized for soybean production in the Midwest USA. At maturity, plots were bulk harvested and threshed and seeds transported to a long-term seed storage room at Sears Plant Growth Facility (University of Missouri, Columbia, MO).

### Amino acid analysis

Amino acid analysis of high and low sulfur accumulating soybean PI lines was performed at the University of Missouri Agriculture Experiment Station Chemical Laboratories, University of Missouri, Columbia, MO utilizing the official AOAC method (AOAC International, Official Method 982.30 E(a,b,c)). For each soybean PI line, seeds from ten randomly selected plants were harvested and polled together. The pooled seed samples were ground to a fine powder with a mortar and pestle. Two hundred milligrams of finely ground soybean seed powder were used for amino acid analysis. Two independent replicates of each PI lines were subjected to hydrolysis for 16 h at 110 °C in 6.0 N HCl. Following this step, amino acids were separated on a Beckman 6300 Amino Acid Analyzer (Beckman Instruments, Fullerton, CA, USA) equipped with a high-performance, cation exchange resin column.

### Protein extraction

For each soybean PI line, seeds from ten randomly selected plants were harvested and polled together. Pooled dry seeds of high and low sulfur accumulating soybean PI lines were ground to a fine power with a mortar and pestle. Total seed protein was obtained following direct extraction with SDS-sample buffer^42^. Briefly, 10 mg of seed powder was extracted with 1 mL of sodium dodecyl sulfate (SDS) sample buffer (62.5 mM Tris–HCl, 2% SDS, 10% glycerol, 30 nM Bromophenol Blue, pH 6.8, 2% (v/v) β-mercaptoethanol) on a vortex mixer for 10 min at room temperature followed by boiling at 100 °C for 5 min. Then, the seed samples were subjected to centrifugation at 15,800×*g* for 10 min and the resulting clear supernatant was saved and designated as total seed proteins.

To obtain protein fraction that is enriched in Bowman-Birk protease inhibitor (BBI) and leginsulin, 50 mg of seed powder was placed in 2 ml plastic tubes. To this 1 mL of 50% isopropanol^30,34^ was added and placed on a vortex mixer for 15 min at room temperature. The slurry was centrifuged at 15,800×*g* for 10 min and 500 µL of the clear supernatant was placed in a 2-mL. To this, 1.5 mL acetone was added and placed at − 20 °C for 15 h. Precipitated proteins were recovered by centrifugation at 15,800×*g* for 10 min. The resulting pellet was air-dried and resolved in 200 µL of 1X SDS-sample buffer.

### One-dimensional gel electrophoresis

SDS-PAGE was conducted using the Mini250 apparatus (GE Healthcare, Piscataway, NJ, U.S.A.). Seed proteins were separated on 13.5% gels while BBI and leginsulin were resolved in 15% gels. Separation was achieved with a constant 20 mA per gel and a typical run time of 1.2 h. Resolved proteins were detected by placing the gels overnight in 0.1% Coomassie Blue R-250 staining solution.

### Immunoblotting analysis

Soybean protein samples resolved on 15% SDS-PAGE were electrophoretically transferred to nitrocellulose membranes for an hour. Following this, the nitrocellulose membrane was incubated with 3% dry milk powder dissolved in Tris-buffered saline (TBS; pH 7.5) for 1 h at 25 °C with gentle shaking. Polyclonal antibodies raised against soybean BBI, leginsulin and lunasin have been previously reported.^30,34,41^ Soybean glycinin 7- (Gy7) specific antibodies were raised in rabbits to *Escherichia coli* expressed Gy-7 proteins^42^. All primary antibodies were used at 1:10,000 dilution in TBS containing 3% dry milk powder for 15 h. Nonspecific binding was eliminated by washing the membrane four times (10 min each wash) with TBS containing 0.05% Tween-20 (TBST). Specifically bound antibodies were detected by incubating the nitrocellulose membrane with 1:20,000 of goat anti-rabbit IgG − horseradish peroxidase conjugate antibody (Bio-Rad) for 1 h. Then, the membrane was washed three times in the same TBST noted above. Immunoreactive polypeptides were visualized by incubation of the membrane with an enhanced chemiluminescent substrate (Super Signal West Pico Kit; Pierce Biotechnology, Rockford, IL). Two technical replicates was performed for the immunoblot analysis.

### Measurement of trypsin and chymotrypsin inhibitor activity

Trypsin inhibitor activity in different soybean cultivars was measured following the procedure described by Kim and Krishnan^43^. Briefly, finely ground soybean seed powder (20 mg) was extracted with 1 ml of 10 mM NaOH on a vortex mixer for 10 min. The slurry was centrifuged at 16,000*g* for 10 min and the resulting supernatant was saved and used for the enzyme assay. Soybean seed extract, which resulted in 30–70% trypsin inhibition range, was added to a 1.5 mL Eppendorf tube containing 200 µl of assay buffer (50 mM-Tris–HCL, pH 8.2 and 20 mM CaCl_2_), 500 µl of BAPNA (0.4 mg/ml), and 200 µl of trypsin (20 ug/ml). The reaction was carried out for 10 min at 37 C, terminated by the addition of 200 µl of 30% acetic acid and absorbance at 410 nm was measured. Trypsin units inhibited (TUI) was calculated as outlined earlier^43^. Trypsin inhibitor activity was expressed as TUI per mg of sample. The results are expressed as the mean ± SD from three biological replicates.

Seed extract for chymotrypsin inhibitor activity was obtained following the procedure described above. Soybean seed extract, which resulted in 30–70% chymotrypsin inhibition range, was added to a 1.5 mL Eppendorf tube containing 900 µl of assay buffer (100 mM-Tris–HCL, pH 8.0), 8 µl of α-chymotrypsin (Sigma-Aldrich Company) dissolved in 1 mM HCl solution (0.1 mg/ml) and seed extract (8–10 µl) and incubated at 37 C for 5 min. Following this step, 80 µl of AAPF (1 mg/ml) was added and incubated for another 10 min at 37 C. The reaction was terminated by the addition of 500 µl of 30% acetic acid and absorbance at 410 nm was measured. Chymotrypsin inhibitor units were calculated as the amount of inhibitor that reduced the absorbance at 410 nm by 0.1 optical density. The results are expressed as the mean ± SD from three biological replicates.

### Statistical analysis

Results are reported as mean ± standard deviation for at least three replicates except when indicated otherwise. We used the FitYbyX function of JMP16 (SAS Institute Inc., Cary, NC, USA) to examine the relationships between sulfur content and amino acid or trypsin or chymotrypsin inhibitor activities, and to plot linear trends among these data. We set the significance level at α = 0.05 for all our analyses.

### Supplementary Information

Supplementary Figures.

## Data Availability

The data generated in this study is available from the corresponding author on reasonable request.
