# Supplementary material for: Elemental sulfur concentration can be used as a rapid, reliable, and cost-effective predictor of sulfur amino acid content of soybean seeds
Source: Sci Rep. 2024 Feb 7;14:3093. doi: 10.1038/s41598-024-53590-3 (PMC10850096; doi:10.1038/s41598-024-53590-3)

Supplementary Information

Elemental sulfur concentration can be used as a rapid, reliable, and cost-effective predictor of sulfur amino acid content of soybean seeds

Wonseok Kim[a], Sunhyung Kim[a], Thomas P. Mawhinney[b], Hari B. Krishnan[a,c,*]

[a]Division of Plant Science and Technology, University of Missouri, Columbia, MO, 65211, United States

[b]Department of Biochemistry, University of Missouri, Columbia, MO, 65211, United States

[c]Plant Genetics Research Unit, USDA, Agricultural Research Service, Columbia, MO 65211, United States

[*]Corresponding author's E-mail address: hari.b.krishnan@usda.gov

Supplemental File 1.

Figure 2A. Uncropped digital image used for creating Figure 2A. The empty lane between the high sulfur lines and the lower sulfur lines was deleted in presenting Figure 2A.

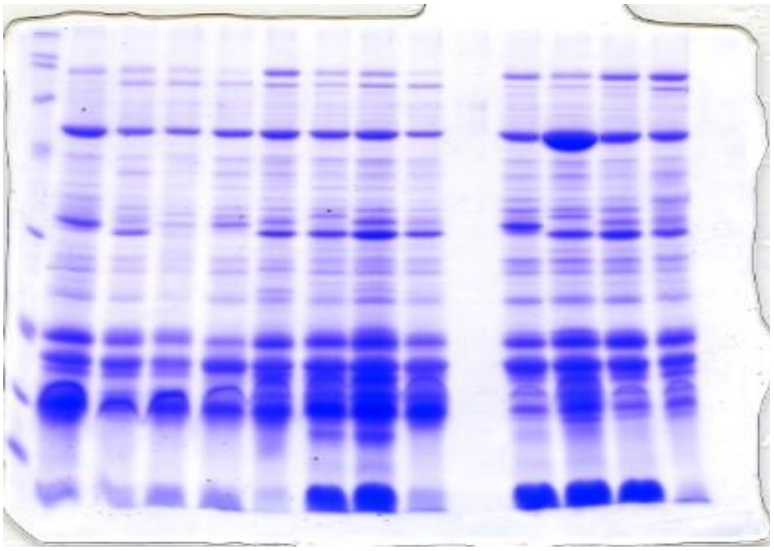

Figure 2B. Uncropped X-ray film image used to create Figure 2. The empty lane between the high sulfur lines and the lower sulfur lines was deleted in presenting Figure 2B.

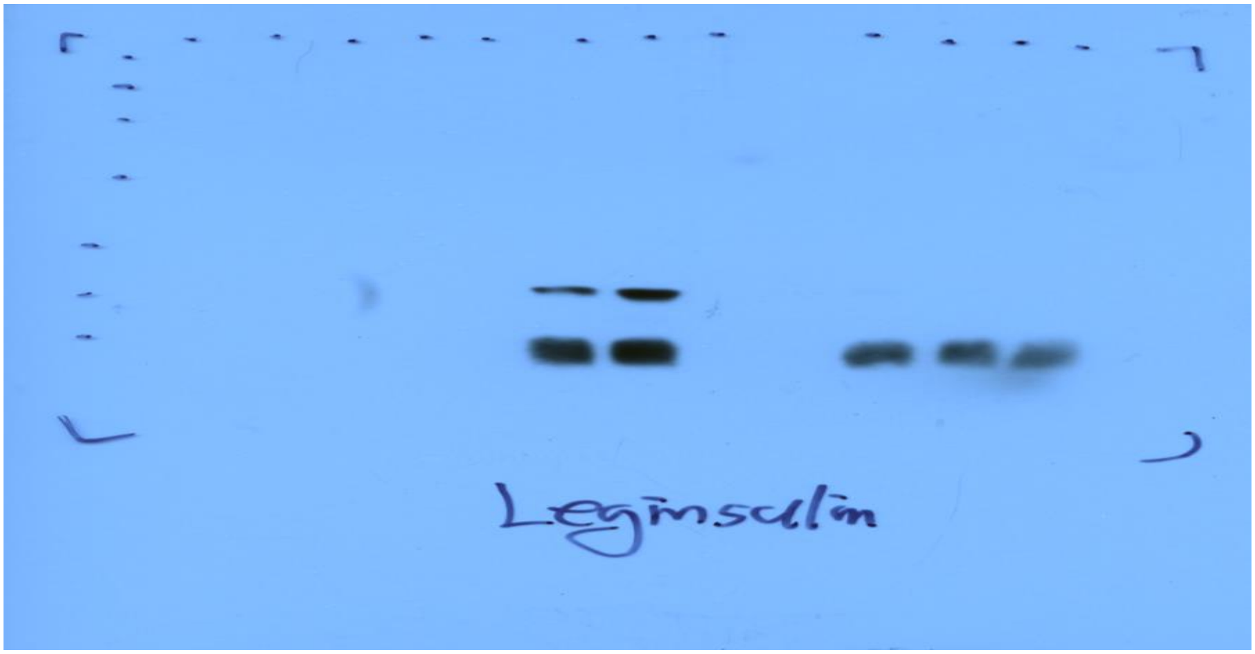

Figure 3A. Uncropped X-ray film image used to create Figure 3A. Only lanes relavant to our study is included in creating figure 3A.

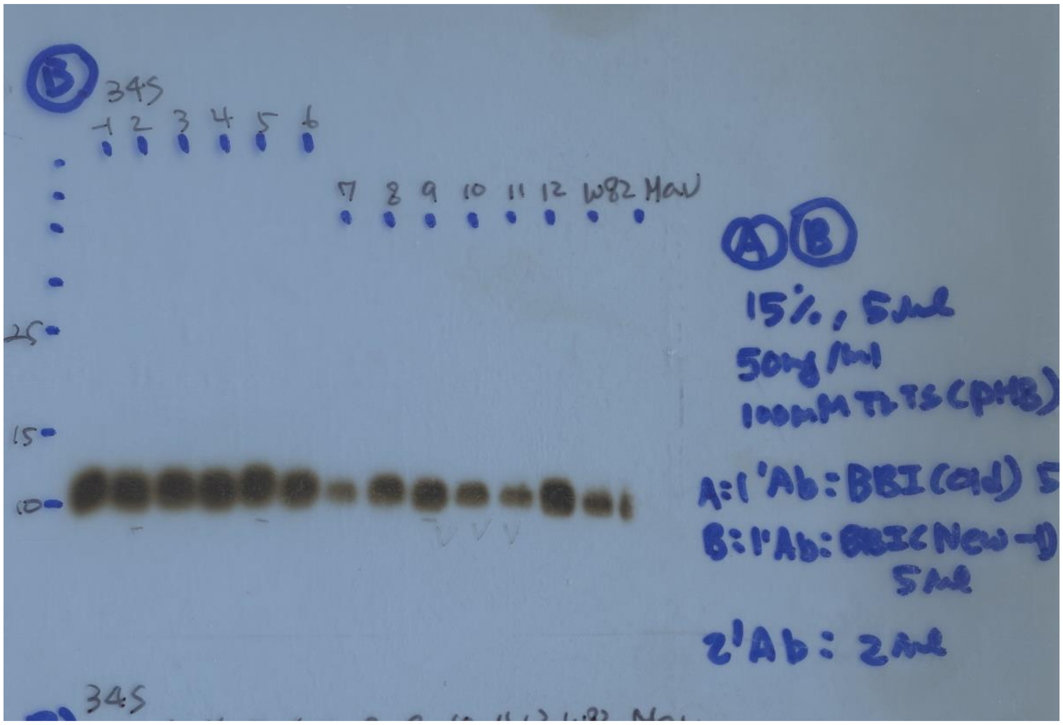

Figure 2B. Uncropped X-ray film image used to create Figure 3B.

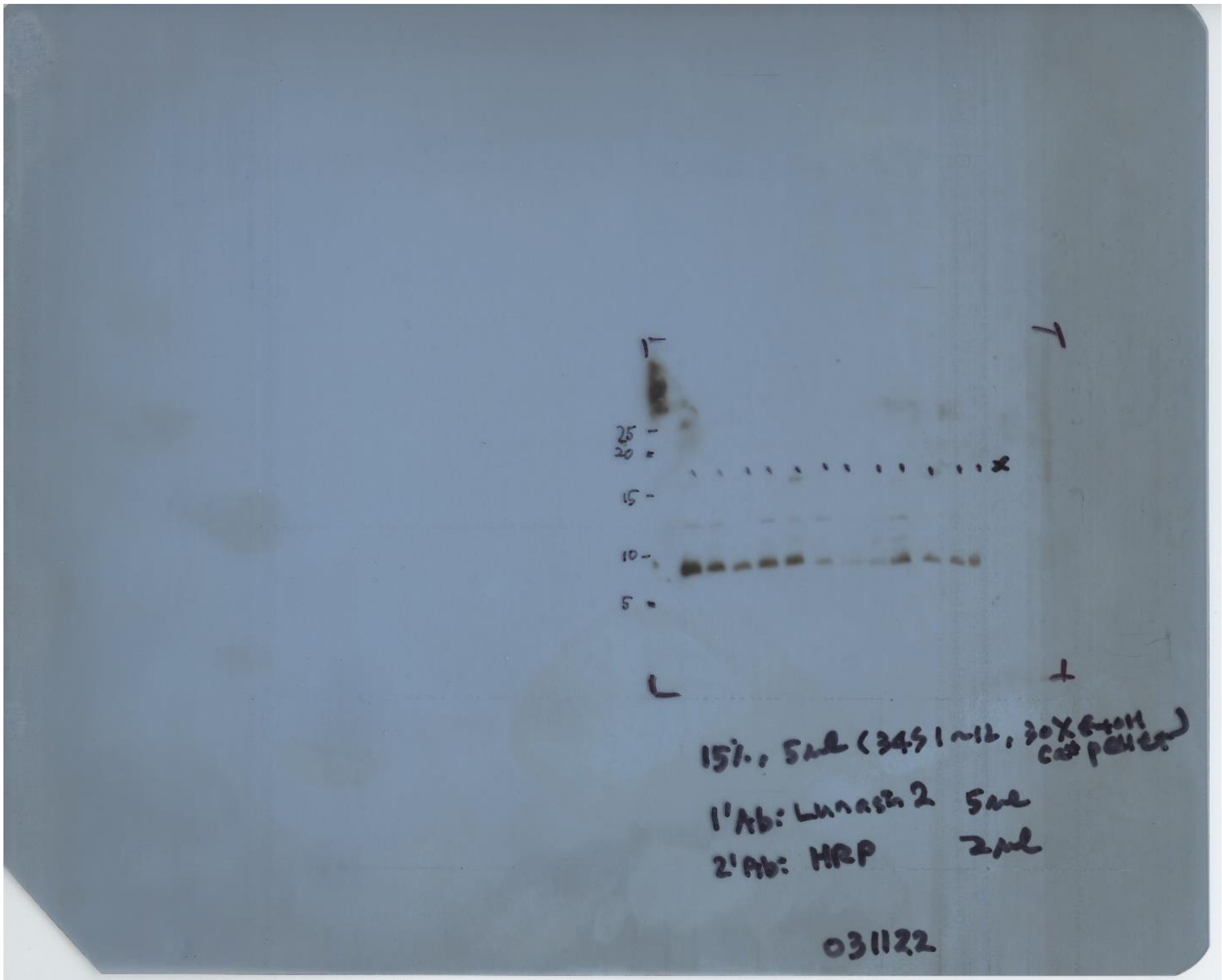

Figure 2B. Uncropped X-ray film image used to create Figure 3C. Only lanes relevant to our study is included in creating figure 3C.

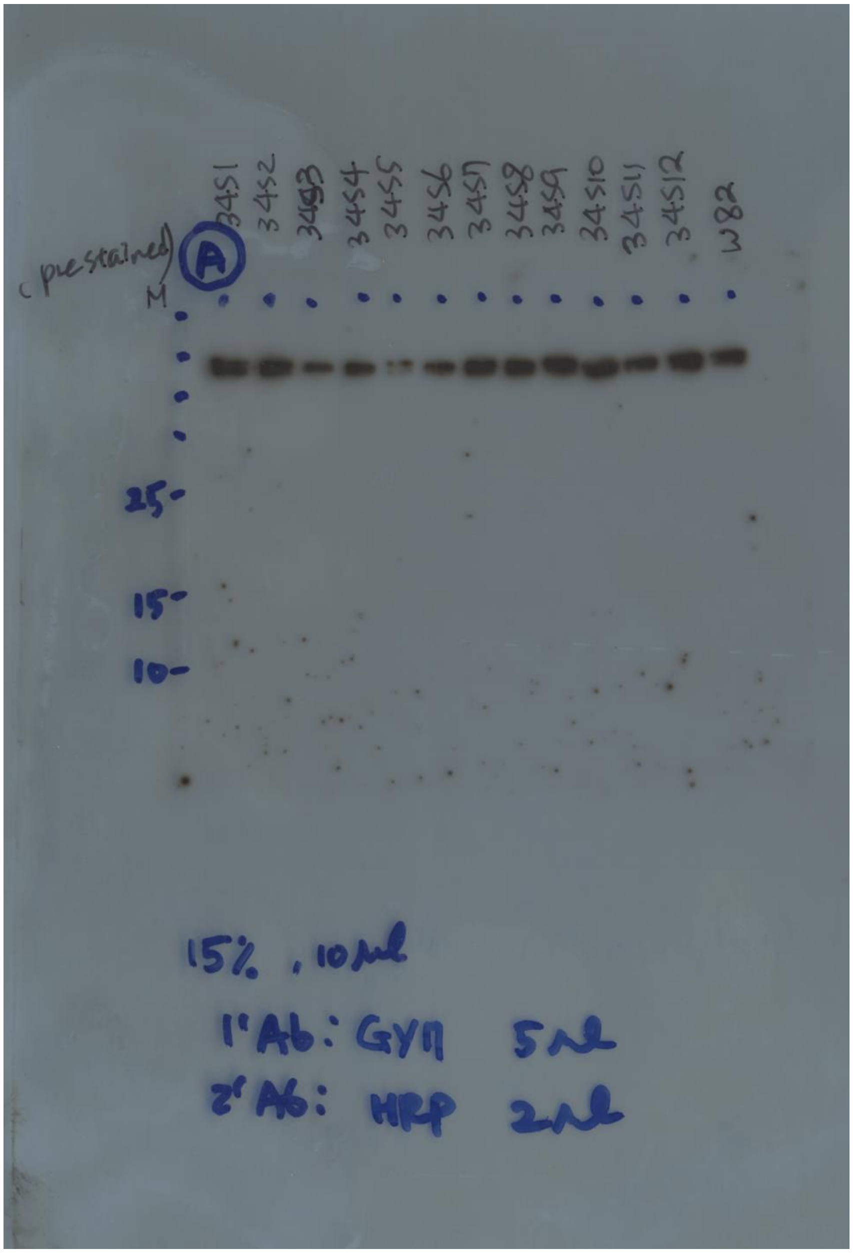

Supplement: Supplementary file 1 — Supplementary Figures. [file 41598_2024_53590_MOESM1_ESM.pdf]
